# Intravesical foreign object: A case report of autoerotism

**DOI:** 10.1016/j.ijscr.2020.11.079

**Published:** 2020-11-19

**Authors:** Albert Ivan Simangunsong, Sawkar Vijay Pramod

**Affiliations:** Urology Department, Hasan Sadikin Academic Medical Center, Universitas Padjajaran Bandung, Jl. Pasteur No. 38, Bandung, Jawa Barat, 40161, Indonesia

**Keywords:** Intravesical foreign bodies, Bladder foreign body evacuation, Case report

## Abstract

The lower urinary tract's foreign body is an uncommon condition, with the bladder being the most common site. Most foreign bodies are self-inserted via the urethra due to exotic impulses, psychometric problems, or sexual curiosity. We present a case of a 39 years old male with a lower urinary tract and abdominal pain. An abdominal CT Scan showed a sharp metal in the bladder. The patient underwent a successful extraction of a foreign body with biopsy forceps transurethrally. Foreign bodies in the bladder represent a urological challenge that requires prompt management. Treatment is determined by size, location, and shape. Minimally invasive procedures are recommended to prevent bladder and urethral injuries. However, surgical treatment should be performed if further injuries are expected. The suspected history and presenting symptoms are crucial and could lead to further investigations. Extraction should be carefully performed to minimize bladder and urethral injury. Gentle endoscopic management is the primary treatment with a high success rate. This case report aims to describe a case of the longest 12 cm sharp metal foreign material inserted to the bladder in South East Asia along with the management and subsequent condition to provide a better insight in managing future unusual cases.

## Highlights

• Foreign body located in the lower urinary tract is uncommon, with bladder being the most common site.
• Most of them are self-inserted via the urethra as the result of psychometric problems, or sexual curiosity.
• Treatment of foreign bodies is determined by their size, location, shape, and mobility.
• In most cases, minimally invasive procedures are recommended to prevent bladder and urethral injuries.
• Surgical treatments are necessary if endoscopic removal fails or further injuries take place.

## 1 Introduction

Foreign body located in the lower urinary tract is an uncommon condition, with the bladder being the most common site. Most foreign bodies are self-inserted via the urethra due to exotic impulses, psychometric problems, or sexual curiosity [1]. If the foreign body is not removed, it may cause pelvic pain, hematuria, retention, and secondary stones. Diagnosis of these foreign bodies can be made by clinical history taking, physical examination, and imaging studies. The treatment is determined by their size, location, shape, and mobility [2]. In most cases, minimally invasive procedures are recommended to prevent bladder and urethral injuries.

However, in some cases, surgical treatment is necessary if endoscopic removal fails or further injuries are to be expected due to the endoscopic procedures.^2^ Al-Heeti et al., in a retrospective study on foreign bodies in the urinary bladder during a period of 10 years in a teaching hospital, only reported 21 cases, of which the most common cause is iatrogenic (42.9 %) followed by self-insertion (33.3 %), migration from outside the bladder (14.3 %) and external trauma (9.5 %) [3]. As far as we know, such research has not been done yet in Indonesia, where mostly sporadic case reports have been published. We describe a case of unusual foreign body insertion by a schizophrenic male, which we managed with cystoscopy.

## 2 Case Presentation

A 39 years old male presented to the emergency department with lower urinary tract symptoms and lower abdominal pain. The symptoms began one week ago and had progressively worsened. Due to the unbearable pain, he was brought to the emergency department by his family. He had previously been diagnosed with schizophrenia and was since cared for by his close relatives. He had no previous history of foreign body insertion into the urethra. Family history was noncontributory. The patient had a surgical midline scar resulting from a laparotomy exploration surgery five years ago due to perforated appendicitis. The patient’s careful history revealed that he had inserted a long, sharp metallic nail into his urethra, which he did not report to his caretaker before the onset of abdominal pain. Fortunately, he did not attempt to remove the nail by inserting other foreign bodies.

Physical examination showed a bruised lower left abdomen. The nail was not immediately palpable in the proximal urethra. Hence we speculated that it might have passed into the bladder. There were no signs and symptoms of perforated hollow organ or ileus. Laboratory findings revealed microscopic hematuria and pyuria in routine urine analysis. Complete blood count only showed slightly increased leukocytes. Electrolyte profiles and renal function were normal. Urinary bladder USG showed a foreign body in the bladder. Thoraco-abdominal X-ray confirmed that there were no perforated organs. An abdominal CT Scan showed a hyperdense lesion in the urinary bladder. We found a foreign body inside the bladder and advised surgery for its removal. The patient underwent transurethral cystoscopy to extract the foreign body by using forceps biopsy.

**Fig. 1:**
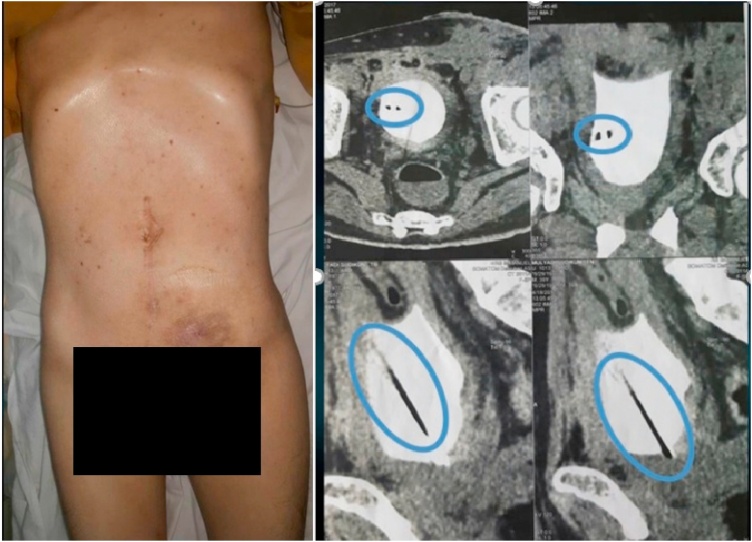
Physical examination revealed bruising on the left lower abdomen and CT-scan showed a solitary sharp-ended foreign body with one end lodged onto the bladder wall.

**Fig. 2:**
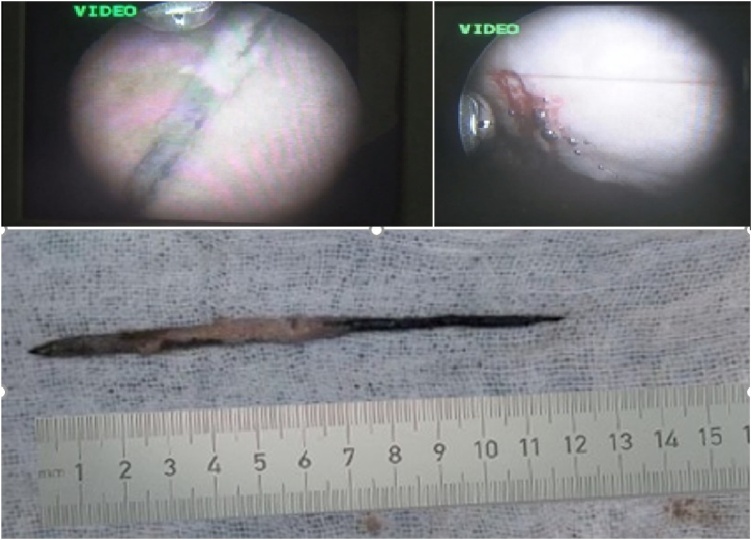
Cystoscopy revealed clear vision of foreign body in the bladder and successful removal of foreign body.

The extraction procedure was carried on by a urologist, an expert in endourology with ten years of experience. Under general anesthesia, the inserted nail was removed by cystoscopy. A dark-colored, straight, metallic sharp object was observed at the bladder cavity’s left lateral aspect. We also identified hyperemia, erosion, and debris in the bladder wall. Fortunately, the foreign body was not stuck onto the bladder wall. However, small bleeding was found on several sites in the inner bladder mucosa caused by the sharp injury. The nail was successfully extracted using cystoscopy and forceps biopsy. A biopsy of the bladder was done to detect any malignancy in the bladder. The nail was measured for 1.5 mm in diameter and 12 cm long with a sharp-ended shape on both ends (Fig. 1, Fig. 2).

The postoperative period was unremarkable, with good operation outcome and no infection. The anatomic pathologic report from the biopsy of the bladder revealed no malignancy in the bladder. The patient was put on an oral prophylactic antibiotics regimen for three days and was scheduled for outpatient follow-up for both the urology and psychiatric departments. Follow-up evaluation in the urology department one week later and one month after the procedure revealed no voiding problems; there was no history of lower urinary tract symptoms. The uroflowmetry examination showed normal results. Foley catheter was removed fourteen days after the surgery, and the patient had no difficulty urinating. Our study has been reported in line with SCARE 18 [4].

## 3 Discussion

The presence of a urinary bladder foreign body has been an interesting topic in representing a management challenge. Most foreign bodies in the lower genitourinary tract are self-inserted via the urethra due to erotic stimulation, psychometric problems, or sexual curiosity. Other causes include iatrogenic during a urological procedure, traumatic aspect, and migration from other organs [1].

Diagnosis is made by history taking and clinical examination. Some patients may show signs of anxiety during sexual history-taking or avoid genital or rectal examination. The clinical presentations of foreign bodies in the urinary bladder may vary from being asymptomatic to complaints of dysuria, hematuria, frequency, poor-stream, suprapubic pain and urinary retention, and chronic pelvic pain. Complications associated with long-standing urinary bladder foreign bodies are recurrent urinary tract infections, stone formation, and urosepsis. Thus, a physician should consider the presence of a foreign body as a differential diagnosis alongside bladder calculi, prostatitis, and prostate hyperplasia when presented with a case of chronic urinary tract infections [2,5].

A plain pelvic radiographic imaging is usually sufficient to locate and identify these objects. CT or ultrasonography is useful as the next workup step. Urethroscopy is the most accurate method for diagnosing foreign bodies in the urinary bladder. In our case, the patient presented to the emergency department before the inserted object calcified or formed stones, therefore contributing the endoscopic retrieval to be more successful [5].

Various options for bladder foreign body treatment include the endoscopic, percutaneous, open, and laparoscopic treatment. The extraction method varies according to the size and mobility of the object inside the urinary bladder. However, endoscopic retrieval is the preferred treatment among urologists. Bansal et al. reported that, of 49 subjects with foreign bodies in the urinary bladder, 33 (67.3 %) were retrieved by cystoscopy, while transurethral cystolitholapaxy was required in 10 patients (20.4 %) [1].

Follow-up of these patients is recommended, as they may develop urethral strictures throughout a long-term follow-up [1].

## 4 Conclusion

Foreign bodies in the urinary bladder represent a urological challenge that requires prompt management. The suspected history and presenting symptoms are crucial and could lead to further investigations. Extraction should be carefully performed to minimize bladder and urethral injury. Gentle endoscopic management is the primary treatment with a high success rate.

## Consent

Written informed consent was obtained from the patient for publication of this case report and accompanying images. A copy of the written consent is available for review by the Editor-in-Chief of this journal on request.

## Conflicts of interest

The authors declare that they have no financial connections with any companies of relevance for this article.

## Declaration of Competing interest

The authors report no declarations of interest.

## Funding

We fund the research all by ourselves.

## Ethical approval

We hereby state that we have the approval from our Hospital Ethical Committee and the patient himself.

## Provenance and peer review

Not commissioned, externally peer-reviewed.

## CRediT authorship contribution statement

**Albert Ivan Simangunsong:** Conceptualization, Methodology, Software, Validation, Formal analysis, Investigation, Resources, Data curation, Writing - original draft, Writing - review & editing, Visualization, Supervision, Project administration, Funding acquisition. **Sawkar Vijay Pramod:** Conceptualization, Methodology, Software, Validation, Formal analysis, Investigation, Resources, Data curation, Writing - original draft, Writing - review & editing, Visualization, Supervision, Project administration, Funding acquisition.
